# What the papers say

**DOI:** 10.1093/jhps/hnaa067

**Published:** 2021-01-17

**Authors:** Ali Bajwa

**Affiliations:** The Villar Bajwa Practice, Princess Grace Hospital, London W1G6PU, UK

The *Journal of Hip Preservation Surgery* (*JHPS*) is not the only place where work in the field of hip preservation may be published. Although our aim is to offer the best of the best, we continue to be fascinated by work that finds its way into journals other than our own. There is much to learn from it so *JHPS* has selected six recent and topical subjects for those who seek a summary of what is taking place in our ever-fascinating world of hip preservation. What you see here are the mildly edited abstracts of the original articles, to give them what *JHPS* hopes is a more readable feel. If you are pushed for time, what follows should take you no more than 10 min to read. So here goes …

## OUTCOMES OF HIP ARTHROSCOPY FOR FEMOROACETABULAR IMPINGEMENT IN CHINESE PATIENTS AGED 50 YEARS OR OLDER

The authors [1] from Daejeon, Korea report a therapeutic prospective case series. A total of 27 patients over the age of 50 years with femoroacetabular impingement (FAI) underwent hip arthroscopy were analysed. The minimum follow-up was 2 years. All patients underwent unilateral surgery. There were 15 male and 12 female patients with a mean age of 57 years (range 50–74). The outcomes were assessed using the visual analogue scale (VAS), the modified Harris hip score (mHHS) and the International Hip Outcome Tool (iHOT‐12).

The post-operative centre-edge angle, the alpha angle and the offset decreased significantly compared with the preoperative measurements. The outcome scores before surgery and at 1 and 2 years after surgery for mean mHHS were 62.19, 86.70 and 87.89; the mean iHOT-12 scores were 30.44, 73.56 and 73.77 and the mean VAS scores were 6.07, 1.93 and 1.59, respectively. There was a significant improvement in the mean mHHS, iHOT-12 and VAS scores at 1 and 2 years after surgery. The mHHS score at 2-year follow-up after surgery was significantly higher than that at 1 year after surgery. One patient with severe acetabular and femoral articular cartilage damage underwent total hip replacement 11 months after index surgery.

The authors concluded that the hip arthroscopy considerably improved hip symptoms and function in Chinese FAI patients who were 50 years or older and did not have severe radiographic osteoarthritis. The conversion to total hip arthroplasty (THA) and complications were reported to be low. Adherence to strict surgical indications and appropriate surgical strategies were considered important in order to lay the foundation for satisfactory post-operative results in elderly patients with FAI.

## IS THERE A GENDER GAP IN OUTCOMES AFTER HIP ARTHROSCOPY FOR FAI? ASSESSMENT OF CLINICALLY MEANINGFUL IMPROVEMENTS IN A PROSPECTIVE COHORT

Flores *et al*. [2] from the United States evaluated the outcomes after hip arthroscopy for FAI based on patient gender in this prospective cohort study. Although patients have experienced significant improvements after hip arthroscopy for FAI, prior studies suggest that women have worse outcomes than men. These previous studies lack comparisons of patient-reported outcome (PRO) scores based on gender with respect to clinical significance measurements, including the minimal clinically important difference (MCID) and patient acceptable symptom state (PASS). In this study, women and men undergoing hip arthroscopy for FAI were prospectively enrolled, and preoperative radiographic and intraoperative findings were collected. Patients completed the following PRO surveys before surgery and 2 years postoperatively: mHHS, hip disability and osteoarthritis outcome score (HOOS) and 12-item short form health survey. Mean scores and percentage of patients reaching MCID and PASS were analysed.

The authors reported that a total of 131 hips were included (72 women, 59 men) in the study. Women had a smaller mean preoperative alpha angle than men (59.1° versus 63.7°, respectively) and lower acetabular cartilage injury grade (6.9% versus 22.0% with grade-4 injury, respectively). Both women and men achieved equivalent significant improvements in PRO scores after surgery (scores increased 18.4–45.1 points for mHHS and HOOS). Women and men both reached PASS for mHHS at similar rates (76.4% and 77.2%, respectively). MCID was also achieved at similar rates between women and men for all scores (range, 61.4–88.9%) except the activities of daily living (ADL) subscale of the HOOS, in which a significantly greater percentage of women reached MCID compared with men (79.2% versus 62.7%). Additional stratification by age group using the median cohort age of 34 years showed no significant differences in PRO improvement based on age group for each gender. The authors concluded that women can achieve clinically meaningful improvements in PRO scores after hip arthroscopy for FAI. Compared with men, women demonstrated equivalent high rates of achieving MCID and PASS at 2 years after surgery.

## MIDTERM-CLINICAL OUTCOMES AFTER HIP ARTHROSCOPY IN MIDDLE-AGED PATIENTS WITH EARLY OSTEOARTHRITIS

In this retrospective cohort study, Lee *et al*. [3] report mid-term results of hip arthroscopy in middle-aged patients with early osteoarthritis. They note that although the number of hip arthroscopies is rapidly increasing in non-elderly patients, outcomes of this procedure in middle-aged patients are not well documented or clearly understood. They evaluate the clinical and radiological outcomes after hip arthroscopy in middle-aged patients with early osteoarthritis. A total of 189 patients with early osteoarthritis of various diagnoses aged 40 years or older, who underwent hip arthroscopy over a 5-year period, were analysed. Clinical outcomes were measured using the mHHS, hip outcome score-activities of daily living (HOS-ADL) and the VAS for pain and range of motion. The radiological outcomes were documented using the Tönnis grade. A minimum 3-year follow-up was employed.

The authors report that the mean preoperative and final mHHS and HOS-ADL improved significantly from 61.2 and 60.6–79.5 and 81.8, respectively, while the VAS pain score decreased significantly from 6.3 to 3.2. Although the mean range of internal rotation and flexion increased from 14.2° and 100.7° preoperatively to 30.4° and 110.6° at 1-year postoperatively, they decreased slightly to 27.4° and 105.4° at the final follow-up, respectively. Eight cases (4.2%) underwent revision arthroscopic surgery and three cases (1.6%) were converted to THA. The authors noted that the patients with early-stage osteoarthritis of various diagnoses achieved improved clinical outcomes. Therefore, using hip arthroscopy in middle-aged patients with early osteoarthritis, it is possible to achieve good surgical outcome.

## RISK FACTORS FOR CONVERSION OF HIP ARTHROSCOPY TO THA: A LARGE CLOSED-COHORT STUDY

The authors [4] from California, USA, evaluated the risk factors for conversion of hip arthroscopy to THA within 2 years in a closed patient cohort. This study was a case series of consecutive hip arthroscopy procedures from September 2008 to November 2018 in the electronic medical record of Kaiser Permanente Northern California. Patients were included with a minimum 2-year follow-up, or if they had conversion to THA within the 2 years (the primary outcome), regardless of follow-up time. Patient characteristics at the time of the index arthroscopy were extracted; characteristics of patients who experienced the outcome event versus those who did not, were compared by use of multivariable logistic regression models and receiver operating characteristic curves.

It was reported that the mean follow-up time was 4.9 years, the mean age was 37.2 years (range 10–88), and 57% were female. During the follow-up period, 82 patients underwent a THA within 2 years of their arthroscopies (5.3%), after a median time of 9 months after the initial arthroscopy. Increasing age was highly predictive of early THA conversion. Although other predictors showed significant bivariable associations with early failure, body mass index (BMI), race, sex and prior arthroscopy did not add meaningful independent predictive information.

The authors concluded that the risk of conversion to THA within 2 years after hip arthroscopy increased substantially with patient age at the time of the procedure. BMI, race, sex, and prior arthroscopy were not important independent predictors of conversion beyond the information contained in patient age.

## RECREATIONAL SPORTS AND INTRA-ARTICULAR HIP INJURIES IN PATIENTS UNDERGOING HIP ARTHROSCOPY FOR FAI

Martinez *et al.* [5] aimed to determine the relationship between recreational sports and intra-articular hip injuries in an active population that had undergone hip arthroscopy for FAI syndrome. They performed a retrospective review of prospectively collected data from patients undergoing hip arthroscopy. Inclusion criteria included patients between 18 and 50 years of age who had participated in recreational sports prior to surgery and had a minimum of a 2-year follow-up. Labral injury was evaluated using the Multicentre Arthroscopic Hip Outcome Research Network classification, and rim chondral injuries were evaluated using the Acetabular Labral Articular Disruptions system. Ligamentum teres tear and psoas impingement were also recorded. Sports were classified as rotational running (soccer, basketball and handball), flexibility (martial arts and dance), asymmetric-overhead (racquet) or endurance (running, swimming and cycling). Primary univariate analysis of sports’ independent associations, demographic characteristics, intra-articular hip injuries and outcomes was performed.

In the final result they included 185 patients with a mean age of 36.7 years. Patients participating in rotational running sports and flexibility sports had a significantly greater proportion of rim chondral injuries than those participating in endurance sports or asymmetric-overhead sports. Ligamentum teres tears were significantly associated with flexibility sports. A total of 84.7%, 67.7%, 67.2% and 71.2% of patients met MCID levels for the mHHS, the hip outcome score questionnaire with ADL, the sports subscale (HOS-SSS) and the iHOT-12, respectively; 94.9%, 66.2% and 62.7% met the PASS for mHHS, HOS-ADL and HOS-SSS, respectively; 86.7%, 48.5%, 47.8% and 32.4% found substantial clinical benefit for mHHS, HOS-ADL, HOS-SSS and iHOT-12, respectively.

The authors highlighted that the rotational running sports were significantly associated with rim chondral injuries. While the flexibility sports were significantly associated with rim chondral injuries and ligamentum teres tears. Athletes participating in these sports are more likely to have intra-articular hip injuries than those in the other sports categories.

## IMPLEMENTATION OF THE OBTURATOR NERVE BLOCK INTO A SUPRA-INGUINAL FASCIA ILIACA COMPARTMENT BLOCK-BASED ANALGESIA PROTOCOL FOR HIP ARTHROSCOPY: RETROSPECTIVE PRE–POST STUDY

In this study, Lee *et al.* [6] note that the effect of supra-inguinal fascia iliaca compartment block (SI-FICB) in hip arthroscopy is not apparent. It is also controversial whether SI-FICB can block the obturator nerve, which may affect post-operative analgesia after hip arthroscopy. They compared analgesic effects before and after the implementation of an obturator nerve block into SI-FICB for hip arthroscopy. They retrospectively reviewed the medical records of 90 consecutive patients who underwent hip arthroscopy from January 2017 to August 2019. Since August 2018, the analgesic protocol was changed from SI-FICB to SI-FICB with an obturator nerve block. According to the analgesic regimen, patients were categorized as group N (no blockade), group F (SI-FICB only) and group FO (SI-FICB with obturator nerve block). Primary outcome was the cumulative opioid consumption at 24 h after surgery. Additionally, cumulative opioid consumption at 6 and 12 h after surgery, pain score, additional analgesic requests, intraoperative opioid consumption, hemodynamic stability and post-operative nausea and vomiting were assessed. Among the 87 patients, there were 47 patients in group N, 21 in group F and 19 in group FO. The cumulative opioid (fentanyl) consumption at 24 h after surgery was significantly lower in the group FO compared with the group N [N: 678.5 (444.0–890.0) µg; FO: 482.8 (305.8–635.0) µg], whereas the group F did not show a significant difference [F: 636.0 (426.8–803.0) µg].

The authors concluded in the light of their findings that implementing an obturator nerve block into SI-FICB can reduce the post-operative opioid consumption in hip arthroscopy.

## CONFLICT OF INTEREST STATEMENT

None declared.
